# Predictive Factors for Morphological and Functional Improvements in Long-Lasting Central Serous Chorioretinopathy Treated with Photodynamic Therapy

**DOI:** 10.3390/biomedicines13040944

**Published:** 2025-04-11

**Authors:** Maciej Gawęcki, Krzysztof Kiciński, Jan Kucharczuk, Monika Gołębiowska-Bogaj, Andrzej Grzybowski

**Affiliations:** 1 Department of Ophthalmology, Pomeranian Hospitals, 84-200 Wejherowo, Poland; krzysztofkg999@icloud.com (K.K.); monikaxvz@gmail.com (M.G.-B.); 2 Dobry Wzrok Ophthalmological Center, 80-392 Gdansk, Poland; 3 Department of Ophthalmology, 10th Military Research Hospital and Polyclinic, 85-681 Bydgoszcz, Poland; jankucharczuk@wp.pl; 4 Department of Ophthalmology, University of Warmia and Mazury, Oczapowskiego 2, 10-719 Olsztyn, Poland; ae.grzybowski@gmail.com; 5 Institute for Research in Ophthalmology, Foundation for Ophthalmology Development, Mickiewicza 24, 61-836 Poznan, Poland

**Keywords:** central serous chorioretinopathy, photodynamic therapy, spectral-domain optical coherence tomography, best corrected visual acuity, subretinal fluid

## Abstract

**Backgrounds**: Photodynamic therapy (PDT) is an established treatment modality in central serous chorioretinopathy (CSCR). The goal of our study was to evaluate the morphological and functional effects of PDT in patients with long-lasting CSCR and determine the related predictive factors for improvement. **Methods**: This retrospective analysis included consecutive patients with chronic CSCR who consented to PDT. The material comprised 98 eyes of 81 patients (67 males and 14 females) with a disease duration longer than 6 months followed for 6 months post treatment. All patients underwent a basic ophthalmological examination including best corrected visual acuity (BCVA) testing and imaging, spectral-domain optical coherence tomography (SD-OCT), and fluorescein angiography. Patients without macular neovascularization (MNV) were subjected to half-dose PDT (3 mg/m^2^) with standard fluence (50 J/cm^2^), guided by indocyanine green angiography. Cases complicated by MNV were subjected to full-dose PDT. **Results**: A morphological response, defined as complete resolution of subretinal fluid, was achieved in 76.29% of cases, and an improvement in BCVA of at least one logMAR line was obtained in 77.53% of cases. The mean BCVA gain was 1.2 logMAR line. All SD-OCT measurements (central retinal thickness, macular volume, mean subfield thickness, subretinal fluid height, and subfoveal choroidal thickness) showed a significant reduction post PDT. A multivariate analysis proved better morphological outcome associations with a younger age and male gender and better visual gains achieved in patients without intraretinal abnormalities. Univariate testing also showed strong relationships between better baseline BCVA and greater functional and morphological improvements, between shorter disease duration and morphological gains, and between the absence of MNV or intraretinal abnormalities and morphological gains. PDT was highly effective in providing a resolution of pigment epithelial detachment (*p* = 0.0004). The observed effect was significantly dependent upon the lower baseline central retinal thickness (*p* = 0.0095). Patients with intraretinal abnormalities or MNV showed moderate improvements post PDT. **Conclusions**: PDT in long-lasting CSCR cases provides good morphological results but generally minor visual gains. Patients’ expectations of significant increases in BCVA after prolonged disease with distinct alterations of the neurosensory retina should be managed.

## 1. Introduction

Central serous chorioretinopathy (CSCR) is a quite common clinical entity affecting predominantly young individuals, usually in their fourth to sixth decades of life [1,2]. CSCR’s pathogenesis is a subject of debate and so are the recommendations for its treatment, so far lacking an international consensus [3]. The chronic form of CSCR, developing in about 85% of cases, is a distinct therapeutic problem as it leads to significant visual loss [4,5]. Among the most effective treatment modalities tried in this form of CSCR are classic laser photocoagulation (CLP) of the leakage point, photodynamic therapy (PDT), subthreshold micropulse laser (SML), and systemic mineralocorticoid inhibitors, such as Eplerenon. The use of CLP is limited only to cases with well-defined leakage points located outside the fovea; thus, it cannot be applied in a significant number of affected patients [6,7]. SML is a cheap treatment modality that proves effective in 40–80% of cases; however, it does not prevent disease recurrences [8]. Mineralocorticoid inhibitors have been widely used for chronic CSCR management in the last decade, with variable success. A few metanalyses showed that their efficacy in providing remission from symptoms is not superior to that of observation or other treatment options [9,10,11]. Recent studies advocate the superiority of PDT over other treatment modalities in CSCR [12,13]. PDT has been used to treat CSCR for more than 20 years [14,15,16]. Although its application has been tested in both acute and chronic cases, it is usually applied in long-lasting CSCR because of its high cost and invasive character. The mechanism of action of PDT is still unclear. The most popular pathogenic theory assumes that transient hypoperfusion at the choriocapillaris, elicited by the stimulation of the photosensitizer verteporfin, results in decreased choroidal congestion and hyperpermeability of the choriocapillaris, enabling the absorption of subretinal and intraretinal fluids [17,18,19]. This mechanism aligns with the pachychoroid theory of CSCR’s etiology, which assumes a significantly increased choroidal thickness in CSCR patients [20,21]. PDT with different doses of verteporfin and laser fluences has been employed to treat CSCR—a full dose (6 mg/m^2^), half dose (3 mg/m^2^), standard fluence (50 J/cm^2^), or reduced fluence (25 J/cm^2^)—with similar results. The reported efficacy of PDT in chronic CSCR is considerable in terms of the improvement in retinal morphology and moderate in terms of functional gain [22,23,24]. These results suggest that baseline factors should be considered when predicting morphological and functional improvements after PDT.

The goal of our study was to evaluate the morphological and functional effects of PDT in patients with long-lasting CSCR and to identify the predictive factors associated with patient improvement. These factors included patient demographics, the disease duration, and morphological and functional features obtained from visual acuity testing and spectral-domain optical coherence tomography (SD-OCT) scanning.

## 2. Materials and Methods

This study was approved by the local bioethical commission of Okręgowa Izba Lekarska (approval number KB-35/2023, dated 16 August 2023). All procedures performed during the study complied with the Declaration of Helsinki.

The retrospective analysis included all consecutive Caucasian patients with chronic CSCR who consented to PDT performed at Dobry Wzrok Ophthalmological Center between June 2022 and October 2024.

The material comprised 98 eyes of 81 patients (67 males and 14 females). All patients were questioned about the duration of their symptoms, and their medical history, including retinal imaging, was carefully reviewed. The study included active CSCR cases that had lasted longer than 6 months and/or had a recurrent course. Before the PDT, most patients had been unsuccessfully treated with classic photocoagulation or subthreshold micropulse laser, anti-VEGF intravitreal injections, and topical or systemic nonsteroidal anti-inflammatory medications.

We adopted the diagnostic criteria for CSCR from the report of the Central Serous Chorioretinopathy International Group [25]. These criteria are based on the results of multimodal imaging, with major criteria including past or present subretinal fluid (SRF) detected on SD-OCT scans, leakage observed on fluorescein angiography (FA), alteration of the retinal pigment epithelium (RPE) visible on fundus autofluorescence (FAF), areas of increased permeability of the choriocapillaris observed on indocyanine green angiography (ICGA), and increased choroidal thickness on SD-OCT scans.

At baseline, all patients underwent a basic ophthalmological examination, including best corrected visual acuity (BCVA) testing, intraocular pressure measurement, slit lamp examination of the anterior segment and eye fundus, SD-OCT scanning, OCT angiography, FAF, and FA, performed either at the center or by the referring practitioner (Visucam 524, 2019, Carl Zeiss Meditec AG, Jena, Germany). ICGA was always performed with the same device in inconclusive cases and before the PDT. The presence of macular neovascularization (MNV) was determined by ICGA and angio-OCT. All procedures except FA and ICGA were also performed at the two follow-up visits: one month and six months post PDT.

SD-OCT measurements were performed with a REVO FC 130 system (2023, Optopol Technology, Zawiercie, Poland). They included the central subfoveal thickness (CST), corresponding to the mean retinal thickness within a central circle of 1 mm diameter; mean subfield thickness (MST), corresponding to the mean retinal thickness in a central circle of 6 mm diameter; macular volume (MV), which is the retinal volume over a central circle of 6 mm diameter in the central RPE plan; maximum subretinal fluid (SRF) height, measured manually; and subfoveal choroidal thickness (SFCT), also measured manually under the foveola. Additionally, the presence of the morphological features of pigment epithelial detachment (PED), neurosensory retina abnormalities (NRAs), including mainly intraretinal cysts, and MNV was also noted for each case before and after treatment.

In patients with CSCR but without MNV, PDT was performed with a half dose of verteporfin (3 mg/m^2^) and a standard laser fluence of 50 J/cm^2^ (Vitra 689, 2020, Quantel Medical, Cournon d’Auvergne, France). Patients with MNV had a full dose of verteporfin (6 mg/m^2^) with standard laser fluence applied during the procedure. The amount of verteporfin required for each patient was calculated from their mass and height. The range of irradiation performed with a 689 nm laser was determined by ICGA examination. The laser spot size was adjusted to cover the entire area of choroidal hyperpermeability determined on the photographs obtained from the ICGA fundus camera with a 500 µm margin. The mean value of the spot size employed in the study cohort was 4361 +/− 1122 µm, with median of 4500 µm and quartiles Q_1_–Q_3_ = 4000–4500 µm. In cases with multiple targets, a second irradiation was performed immediately after the first one. The duration of a single irradiation was 83 s, according to standards of the PDT procedure. The same protocol was applied in all patients.

In this study, patients were categorized as responders and non-responders to PDT according to their retinal morphology and visual function. A patient was considered a morphological responder if complete resorption of SRF was observed after PDT, whereas partial resolution of SRF was considered a non-response. A functional responder was defined as a patient whose BCVA improved by at least one line on the logMAR chart after PDT. For the purpose of the statistical analysis, categorization of responders at six months post PDT was considered.

### 2.1. Statistical Analysis

The results of the treatment of chronic CSCR with PDT were analyzed in terms of the changes in the BCVA and SD-OCT parameters after treatment. The groups of responders and non-responders were then compared in terms of their baseline BCVA, SD-OCT measurements, and presence or absence of PED, NRA, and MNV. Both multivariate and univariate analyses were performed to assess the impact of different factors on morphological and functional outcomes of the treatment.

### 2.2. Statistical Procedures

Categorical variables (frequencies) are described as integers and percentages. Measurements are expressed as means, medians, standard deviations, and lower and upper quartiles. The normality of distributions was tested by the Shapiro–Wilk W test. Levene’s test was performed to estimate the homogeneity of variances. A multifactor analysis of variance (ANOVA) with repeated measurements was conducted to assess the dynamics of normally distributed numerical variables and their differences between the study groups. General estimating equations were applied for non-normally distributed variables. A binary logistic regression model was fitted in order to estimate the odds ratios and their significance for dichotomous explained variables. All multivariate procedures were controlled for the participants’ age, gender, disease duration, and comorbidities. A level of *p* < 0.05 was considered statistically significant. All procedures were carried out using Statistica^TM^ release 13.3 (TIBCO Software Inc., Palo Alto, CA, USA).

## 3. Results

The mean age of the patients was 48.78 ± 10.22 years (Me = 47, Q_1_–Q_3_ =43–54) and the mean disease duration was 63.35 ± 75.31 months (Me = 48, Q_1_–Q_3_ = 20–100). The BCVA and morphological parameters measured by SD-OCT significantly improved during the 6-month follow-up period (Table 1). The effect of SRF height and CST reduction after the PDT was enhanced after the first follow-up visit at 1 month and proved greater at 6 months of follow-up. The BCVA improvement at 6 months was no different from the functional effect at 1 month post treatment. The relationships between the occurrence of morphological and functional effects and baseline qualitative and quantitative features are provided in Table 2 and Table 3. Generally, morphological and functional improvements were noted in similar percentages of patients. Morphological improvements were noted significantly more frequently in male patients in both univariate and multivariate analyses. Functional improvements were poorer in cases with neurosensory retina abnormalities for both of these statistical tests. The univariate analysis showed poorer morphological responses in patients with NRAs or MNV as well. An analysis of morphological and functional responses according to baseline BCVA showed significant differences in the univariate analysis: patients with better baseline visual acuity responded better to treatment and had a tendency for higher visual gains (Table 2). Specifically, BCVA > 0.6 logMAR showed similar percentage of morphological responses and failures, while only one eye with BCVA ≤ 0.2 logMAR did not show any improvement post PDT. Morphological improvements were also strongly associated with a younger age, as proved in both univariate and multivariate tests. The univariate analysis showed higher percentages of morphological resolution in patients with shorter disease durations.

An improvement in the BCVA was noted in 69 eyes (77.53%), with 35 eyes (39.33%) improving by ≤0.1 logMAR and 34 eyes (38.20%) by more than 0.1 logMAR. BCVA stabilization was noted in 11 cases (12.36%), and deterioration of vision occurred in 9 eyes (10.11%).

The BCVA gain, over the 6-month studio period, was meaningfully and positively related to its baseline value (*p* = 0.0072). The multifactor model used did not show any other statistically significant relationships with age (*p* = 0.6286), disease duration (*p* = 0.3276), choroidal thickness (*p* = 0.6990), or retinal thickness (*p* = 0.6299).

The multivariate analysis results for changes in PED and NRAs during the six-month follow-up are provided in Table 4. The prevalence of PED significantly decreased over the 6-month study period (*p* = 0.0004). The multivariate model revealed that the observed effect was statistically significantly dependent upon the baseline CST (*p* = 0.0095): the smaller the CST at baseline, the more pronounced the improvement at 6 months post PDT.

The prevalence of NRAs fluctuated yet did not change significantly over the 6-month study period in the multivariate model (*p* = 0.6973).

**Figure 1:**
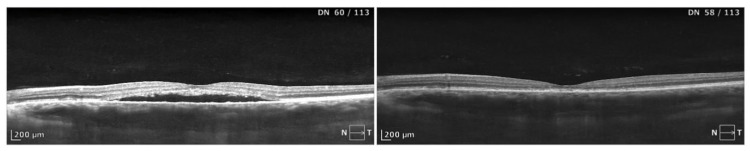
A case of CSCR morphologically presenting with sole subretinal fluid and granulations at the border of the fluid cavity. Complete resolution of the SRF after half dose-PDT was noted at 1 month. Stabilization of the BCVA was noted without improvement at the level of 0.4 logMAR. Significant thinning of the retina can be observed in the macula, which explains the lack of functional gain.

**Figure 2:**
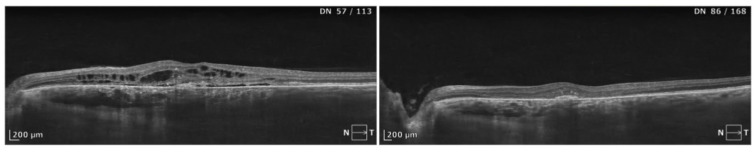
A complex case of CSCR lasting for approximately 36 months, treated with half-dose PDT. Distinct alterations in the neurosensory retina in the form of intraretinal cysts are the predominant morphological feature. A trace of subretinal fluid is also noted. A post-PDT SD-OCT scan revealed considerable resolution of the subretinal and intraretinal fluid. An improvement of 0.1 logMAR was noted post treatment.

**Figure 3:**
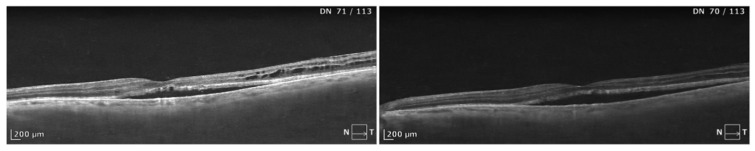
A failure in morphological response after PDT. SD-OCT scans show the case lasting for about 7 years with prominent subretinal fluid and intraretinal cysts. A slight depression of the RPE/choroid line is noted, together with a relatively small choroidal thickness. The amount of SRF was not reduced after PDT, although resolution of the intraretinal cysts was observed. The patient reported a minor improvement in their quality of vision, without gains noted in the visual acuity chart.

**Figure 4:**
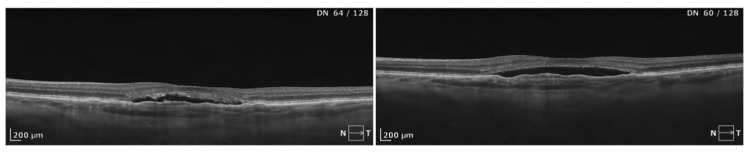
A lack of response to full-dose PDT in a case of chronic CSCR complicated by MNV. At the level of the RPE, characteristic undulation is visible. An increased choroidal thickness was also noted in this case (>400 µm). No reduction in SRF or decrease in the size of sub-RPE MNV was observed. Functional gains were not observed after treatment. The BCVA remained at the level of 0.5 logMAR. The patients remains under close follow-up every 3–4 months. A deterioration in visual acuity was not noted despite the stable morphological picture with MNV and SRF.

Figure 1, Figure 2, Figure 3 and Figure 4 present different responses to PDT in patients with long-term CSCR on the SD-OCT scans.

## 4. Discussion

The results of our study indicate the efficacy of PDT in chronic CSCR. Morphological and functional responses were observed in more than 70% of cases. However, the functional gain was generally modest and seldom exceeded one logMAR line. An improved retinal architecture post PDT was observed with the resolution of SRF and reductions in the central subfoveal thickness, mean subfield thickness, and macular volume. Moreover, a significant decrease in choroidal thickness was noted, which, according to the pachychoroid theory, should be the main cause of therapeutic success. Morphological effects were enhanced at six months post PDT compared to one month, with further reductions in the CST and height of SRF. PDT was also effective in providing resolution of PED. This morphological outcome was achieved in most patients and resulted in visual gains. Such a relationship was not true for cases with intraretinal abnormalities or MNV, the presence of which was linked to a poorer response to PDT.

We revealed strong associations between the response to PDT and different baseline factors in a multivariate analysis. Female gender was linked to a poorer morphological response with OR = 0.14; thus, in our cohort, women had 86% smaller odds of achieving the resolution of SRF as compared to the examined men. Poorer functional gain was associated with the presence of neurosensory retina abnormalities (OR = 0.16). These patients had smaller chances of achieving BCVA improvement, by 84%. Lastly, a longer duration of disease effected a worse morphological response (OR = 1.08). The odds of losing such effects were 8% higher with every year of the patient’s age.

A univariate analysis also proved other associations of baseline factors with PDT effects, among which baseline visual acuity and disease duration seem to be the most important. Patients with relatively good initial BCVA responded very well to treatment, in both morphological and functional respects. Cases with high BCVA (≤0.2 logMAR) practically always presented with significant improvements. On the other hand, low BCVA (>0.6 logMAR) was associated with a poor morphological response but not necessarily a lack of functional improvement. At this level, patients demonstrated minor visual gains after, for example, a reduction in the CST or intraretinal fluid. Longer durations of symptoms were strongly related to poorer morphological responses post PDT.

The results depicted above have the potential to influence the clinical approach to CSCR cases and enable us to better predict treatment outcomes. Patients burdened with the abovementioned factors should be advised to temper their expectations for perfect outcomes after PDT.

In many cases in this study, the resolution of SRF and the restoration of the retinal architecture did not result in significant functional improvement. The median CST after PDT was only 227.00 µm, compared with the 238 µm reported for healthy subjects by the same group [26]. This finding explains the lack of significant visual recovery observed in many patients. The loss of retinal layers due to the prolonged presence of SRF has also been concluded in many studies [27,28,29,30]. It appears that in long-lasting cases, the damage to photoreceptors is sufficient to preclude increased visual acuity.

In the present study, the duration of the disease was long, extending over years rather than months. In this context, the morphological recovery of the retina in the study cohort is highly noteworthy. This is in contrast to the modest functional improvement observed, of about one logMAR line on average. Because the main goal of any ophthalmological treatment is to increase the BCVA, such a result should be considered as moderately successful. It also suggests the importance of early treatment of CSCR, before the loss of retinal cells occurs. In contrast, improvements are less likely to occur after 12 months of CSCR, as reported in a recent study [13].

Interestingly, the morphological response to PDT was poorer in female patients. Note, however, that MNV was present in 5 out of 17 female eyes (almost 30% of the female cohort), compared with 8 out of 81 male eyes (about 10%). Cases of CSCR with MNV seldom respond well to PDT, as observed in other studies [31,32]. The high percentage of eyes with MNV in the female subgroup may partially explain the poor outcome.

Our results should be considered in the context of other reports. Significant morphological recovery after PDT with a minor visual gain has frequently been reported [33,34,35], with an increase in BCVA exceeding one logMAR line noted in a minority of studies [36,37]. Thus, our results are consistent with those of other reports. Table 5 summarizes the prognostic factors analyzed in recent studies of the response of chronic CSCR to PDT.

Younger age has also been reported as a positive prognostic factor post PDT except in a few studies, with Park et al., van Rijssen et al., and Nakamura et al. noting a better response to PDT in younger patients [37,40,42]. Van Rijssen et al. observed a higher percentage of male non-responders than female non-responders to PDT, in contrast to our results, although their sample size was small [40].

Other authors have identified morphological features not analyzed in this study as predictors of the response to PDT. Mirshahi et al. reported a lower response rate in cases with hyperreflective foci in the retina before treatment [43]. Arrigo et al. analyzed the effect of the same biomarker located in the choroid and obtained a similar outcome [41]. Van Rijssen et al. noted a worse response to PDT in patients with diffuse leakage on FA and without intense hyperfluorescence on ICGA [40]. This finding was confirmed by Ozkaya et al., who reported a better response in patients with midphase hypercyanescence on ICGA [48]. These reports address issues not considered in our study, in which we focused on features that could be reliably quantified, although the scleral thickness was not analyzed.

We did not find a correlation between basic SD-OCT measurements and the response to PDT. However, some studies have reported greater improvements in patients with a thicker outer nuclear layer and an intact external limiting membrane, ellipsoid zone, and interdigitation zone [38,43,44,45,46]. These factors were not examined in our study, as we believe that they are difficult to evaluate in the presence of retinal edema, which can bias and distort measurements.

Wakatsuki et al. and van Rijssen et al. also reported an association between a poorer response in patients with a lower baseline BCVA and a longer duration of CSCR, consistent with our results [38,40].

In the context of the published research, our study is noteworthy for its clear definition of non-responsiveness to PDT in both anatomical and functional aspects, as well as its simultaneous analysis of prognostic factors for optimal treatment results in these two areas. In particular, the multivariate analysis provided significant results that could influence current clinical practice.

In contrast, most previous studies only focused on morphological success (dry macula). Such an approach provides only partially reliable conclusions, as many patients exhibiting a perfect anatomical response do not have improved visual function. Moreover, suboptimal morphological responses with reduced intraretinal cysts and reduced SRF can result in minor but observable functional improvements.

### Study Limitations

The main study limitation is the lack of a control group. On the other hand, the very long durations of symptoms in the CSCR cases included in this study practically preclude the possibility of spontaneous remission. Thus, we believe that this limitation does not significantly bias the conclusions of the study.

This study focused on SD-OCT measurements for the morphological description of CSCR patients owing to their quantifiability. However, other diagnostic modalities that were not included in our analysis, such as FA and FAF, also provide valuable information on the condition of the RPE.

Another limitation is the relatively small number of female patients in the study cohort, which may have biased the analysis of the outcomes of PDT in female patients. Moreover, our cohort only consisted of Caucasian patients, and an association between response to PDT and race in CSCR was not ruled out in earlier studies [50].

## 5. Conclusions

The data obtained from this study demonstrated the efficacy of PDT in long-lasting cases of CSCR, although the observed improvements were mainly morphological rather than functional. Patients’ expectations of significant increases in BCVA after prolonged disease should be managed. Better morphological response was associated with male gender, shorter disease duration, and younger age, while functional improvements were greater in the absence of intraretinal abnormalities and in cases of better initial visual acuity. SD-OCT measurements of retinal and choroidal thicknesses before treatment cannot solely guide eligibility for PDT in chronic CSCR.

## Figures and Tables

**Table 1:** Descriptive statistics for treatment-related changes in numerical traits over the 6-month study period (n = 98 eyes).

Analyzed Trait	Follow-Up	Statistical Parameter *				*p* Value **
		*M*	*SD*	*Me*	*Q*_1_–*Q*_3_	
BCVA [logMAR]	Before treatment	0.46	0.37	0.40	0.20–0.60	<0.0001
	At 1 month	0.35	0.34	0.30	0.10–0.50	0.6547
	At 6 months	0.34	0.38	0.20	0.10–0.50	<0.0001
CST [µm]	Before treatment	327.26	82.06	307.00	269.00–396.00	<0.0001
	At 1 month	249.41	80.63	226.00	194.00–275.00	<0.0001
	At 6 months	244.10	76.34	227.00	195.00–264.00	<0.0001
MST [µm]	Before treatment	302.82	29.46	298.00	283.00–312.00	<0.0001
	At 1 month	281.79	26.89	277.00	267.00–294.00	0.1589
	At 6 months	284.49	29.69	280.00	268.00–297.00	<0.0001
MV [mm^3^]	Before treatment	8.54	0.81	8.43	8.01–8.79	<0.0001
	At 1 month	7.97	0.77	7.82	7.54–8.27	0.0779
	At 6 months	8.08	0.90	7.99	7.56–8.47	<0.0001
SFCT [µm]	Before treatment	587.32	110.45	598.00	520.00–660.00	<0.0001
	At 1 month	539.83	102.67	550.00	474.00–603.00	0.8605
	At 6 months	539.09	93.35	548.00	489.00–594.00	<0.0001
SRF, height [µm]	Before treatment	128.26	91.09	120.00	66.00–180.00	<0.0001
	At 1 month	36.95	84.12	0.00	0.00–0.00	0.0184
	At 6 months	30.80	77.76	0.00	0.00–0.00	<0.0001

* Statistical parameters used: M—mean; SD—standard deviation; Me—median; Q—quartile. ** The first *p* value refers to the change observed after 1 month, the second *p* value refers to the change between 1 and 6 months, and the third *p* value concerns the changes over the entire 6-month study period. The models were controlled for the subjects’ age, gender, disease duration, and comorbidities. Abbreviations: BCVA—best corrected visual acuity; CST—central subfield thickness; MST—mean subfield thickness; MV—macular volume; SFCT—subfoveal choroidal thickness; SRF—subretinal fluid. Missing data were case-wise deleted.

**Table 2:** Baseline characteristics of the study cohort by occurrence of improvement observed 6 months after PDT (n = 81 individuals = 98 eyes) (***qualitative traits***).

Analyzed Trait	Morphological Improvement		Functional Improvement		*p* Value *	
	Yes	No	Yes	No	*Univariate*	*Multivariate*
No. of eyes, *n (%)*	74 (76.29)	23 (23.71)	69 (77.53)	20 (22.47)	>0.9999	n/a
Gender, *n (%)*:						
Female	9 (12.16)	8 (34.78)	10 (14.49)	5 (25.00)	0.0127 0.2691	0.0097 OR = 0.14 (95% CI: 0.03–0.62) *p* = 0.1549
Male	65 (87.84)	15 (65.22)	59 (85.51)	15 (75.00)		
MNV, *n (%)*:						
Present	7 (9.46)	6 (26.09)	7 (10.14)	4 (20.00)	0.0409 0.2792	0.8063 0.9306
Absent	67 (90.54)	17 (73.91)	62 (89.86)	16 (80.00)		
PED, *n (%)*:						
Present	14 (18.92)	5 (21.74)	17 (24.64)	2 (10.00)	0.7660 0.2212	0.3554 0.2122
Absent	60 (81.08)	18 (78.26)	52 (75.36)	18 (90.00)		
NRA, *n (%)*:						
Present	8 (10.81)	9 (39.13)	8 (11.59)	8 (40.00)	0.0018 0.0036	0.1547 0.0139 OR = 0.16 (95% CI: 0.04–0.069)
Absent	66 (89.19)	14 (60.87)	61 (88.41)	12 (60.00)		
Baseline BCVA, *n (%)*:						
logMAR ≤ 0.2	24 (32.43)	1 (4.35)	24 (34.78)	1 (5.00)	0.0092 ** 0.0303	0.2747 0.1671
0.2 < logMAR ≤ 0.6	43 (58.11)	16 (69.56)	36 (52.18)	16 (80.00)		
logMAR > 0.6	7 (9.46)	6 (26.09)	9 (13.04)	3 (15.00)		

* The first row: *p* value for morphological improvement; the second row: *p* value for functional improvement. A multivariate binary logistic regression model. ** Paired comparisons: morphological improvement observed when 1 vs. 3 *p* = 0.0093, 1 vs. 2 *p* = 0.0392, 2 vs. 3 *p* = 0.1844; functional improvement observed when 1 vs. 3 *p* = 0.0881, 1 vs. 2 *p* = 0.0261, 2 vs. 3 *p* = 0.6940. Abbreviations: n—integer number, %—percentage, MNV—macular neovascularization; PED—pigment epithelial detachment; NRA—neurosensory retina abnormalities. Missing data were case-wise deleted.

**Table 3:** Baseline characteristics of the study cohort by occurrence of improvement observed 6 months after PDT (n = 81 individuals = 98 eyes) (***quantitative traits***).

Analyzed Trait	Morphological Improvement		Functional Improvement		*p* Value *	
	Yes	No	Yes	No	*Univariate*	*Multivariate*
Age [year], M (SD); Me (Q_1_–Q_3_)	46.63 (8.56); 46 (42–52)	55.52 (12.41); 54 (47–66)	48.49 (8.89); 46 (43–54)	53.15 (12.73); 51 (44–62)	0.0012 0.0824	0.0147 OR = 1.08 (95% CI: 1.02–1.15) 0.3319
Disease duration [month], M (SD); Me (Q_1_–Q_3_)	58.57 (72.30); 30 (20–70)	99.65 (74.87); 96 (48–100)	68.61 (76.79); 48 (20–100)	75.50 (74.84); 60 (27–100)	0.0006 0.3193	0.7329 0.4597
CST [µm], M (SD); Me (Q_1_–Q_3_)	325.06 (79.12); 306.00 (267–401)	334.30 (92.45); 321 (270–396)	323.03 (84.15); 300 (267–377)	334.15 (76.94); 319 (262–403)	0.6397 0.5975	0.8161 0.9125
MST [µm], M (SD); Me (Q_1_–Q_3_)	302.58 (27.27); 299 (285–312)	303.61 (36.32); 297 (273–314)	301.81 (29.19); 297 (285–309)	301.35 (31.06); 303 (273–324)	0.8847 0.9512	0.9153 0.6127
Baseline BCVA [logMAR], M (SD); Me (Q_1_–Q_3_)	0.39 (0.31); 0.35 (0.20–0.50)	0.67 (0,46); 0.60 (0.40–0.80)	0.43 (0.36); 0.40 (0.20–0.60)	0.58 (0.40); 0.50 (0.40–0.60)	0.0008 0.0323	0.0899 0.9566

* The first row: *p* value for morphological improvement; the second row: *p* value for functional improvement. A multivariate binary logistic regression model. Abbreviations: n—integer number, %—percentage, M—mean, SD—standard deviation, Me—median, Q—quartile; MNV—macular neovascularization; PED—pigment epithelial detachment; NRAs—neurosensory retina abnormalities. Missing data were case-wise deleted.

**Table 4:** Changes in the prevalence of PED and NRAs due to PDT over the 6-month study period (n = 98 eyes) in a multivariate analysis.

Morphological Feature	Follow-Up	Statistical Parameter *		*p* Value **
		n	%	
PED	Before treatment	19	19.59	0.0004
	At 1 month	5	5.15	
	At 6 months	4	4.12	
NRA	Before treatment	17	17.53	0.6973
	At 1 month	11	11.34	
	At 6 months	9	9.28	

* Statistical parameters used: n—number; %—percentage. ** The models were controlled for the subjects’ age, gender, disease duration, baseline BCVA, CST, and MST. Abbreviations: PDT—photodynamic therapy; PED—pigment epithelial detachment; NRA—neurosensory retina abnormalities. Missing data were case-wise deleted.

**Table 5:** Factors analyzed in recent studies of the response of chronic CSCR to PDT.

No.	Analyzed Factor	Studies	Finding
1	Disease duration	Wakatsuki et al. 2021 [38], Li et al. 2022 [39], present study	Longer duration associated with poorer morphological outcomes
2	BCVA before PDT	Wakatsuki et al. 2021 [38], van Rijssen et al. 2018 [40], present study	Poorer morphological response at 3 months (Wakatsuki et al.), 2 months (van Rijssen et al.), or 1 month (present study) associated with lower baseline BCVA
3	Age	van Rijssen et al. 2018 [40], Park et al. [37], Arrigo et al. 2021 [41], Nakamura et al. 2022 [42], present study	Older age associated with worse morphological outcomes and non-response
4	Gender	van Rijssen et al. 2018 [40], present study	Anatomical non-response rate greater in male cohort (van Rijssen et al.) or worse anatomical response in female patients (present study)
5	Choroidal abnormalities	Arrigo et al. 2021 [41], Nakamura et al. [42]	Poorer morphological response with hyperreflective foci in the choroid (Arrigo et al.), thicker pretreatment choroidal thickness associated with better morphological response (Nakamura et al.)
6	Presence of MNV	Nakamura et al. 2022 [42], Kamimura et al. 2023 [31], present study	Poorer morphological response and/or recurrence in patients with MNV
7	Retinal thickness, condition of retinal layers, intraretinal abnormalities	Wakatsuki et al. 2021 [38], Mirshahi et al. 2024 [43], Li et al. 2022 [39], Sousa et al. 2019 [44], Son et al. 2024 [45], Yu et al. 2022 [46], present study	Higher CST associated with poorer morphological results at 3 months (Wakatsuki et al.), hyperreflective foci in the retina associated with smaller anatomical improvement (Mirshahi et al.), larger SRF and PED at baseline associated with residual SRF post PDT (Li et al.), thicker ONL associated with better morphological (Sousa et al.) and functional (Yu et al.) responses, intact outer retina correlated with better anatomical response (Sousa et al., Son et al.), foveal atrophy associated with poorer morphological and functional outcomes (Son et al.), smaller morphological improvement in patients with intraretinal abnormalities (present study)
8	Leakage in FA	van Rijssen et al. 2018 [40]	Diffuse leakage over area larger than disc diameter associated with poorer morphological outcomes
9	ICGA findings	Jeong et al. 2024 [47], van Rijssen et al. 2018 [40], Ozkaya et al. 2017 [48]	Larger number of macular vortex veins and vortex vein engorgement associated with poorer morphological outcomes and smaller BCVA improvements (Jeong et al.), lack of intense hyperfluorescence on ICGA associated with poorer morphological outcomes (van Rijssen et al.), better anatomical response in patients with midphase focal hyperfluorescence (Ozkaya et al.)
10	Scleral thickness	Forte et al. 2024 [49]	Increased scleral thickness associated with higher risk of anatomical non-responsiveness

## Data Availability

The original contributions presented in this study are included in the article. Further inquiries can be directed to the corresponding author.
